# Supplementary material for: Characterizing engagement dynamics across topics on Facebook
Source: PLoS One. 2023 Jun 28;18(6):e0286150. doi: 10.1371/journal.pone.0286150 (PMC10306180; doi:10.1371/journal.pone.0286150)
Supplement: S1 File — Moreover, here are provided the figures reporting the correlations between α and LH Score for each topic and the goodness of the fitting procedures. (PDF) [file pone.0286150.s002.pdf]

Supporting Information: Characterizing Engagement Dynamics across Topics on Facebook

Gabriele Etta[1], Emanuele Sangiorgio[2], Niccolò Di Marco[3], Michele Avalle[1], Antonio Scala[4], Matteo Cinelli[1], Walter Quattrociocchi[1,*]

1 Center of Data Science and Complexity for Society, Department of Computer Science, Sapienza Università di Roma
2 Department of Social Sciences and Economics, Sapienza Università di Roma
3 Department of Mathematics and Computer Science, University of Florence, Italy
4 ISC-CNR UoS Sapienza, Rome,Italy

* quattrociocchi@di.uniroma1.it

List of topics employed

Topic Keywords	First Post Date	Last Post Date	Categories
Amyotrophic_lateral_sclerosis	2018-01-02	2021-12-31	Social, Health
DeleteUber	2018-01-01	2021-12-19	Labor, Social
Roy_Moore_sexual_misconduct	2018-01-02	2021-12-13	Human_Rights, Politics, Social
abilene_zoo	2018-01-08	2022-01-01	Art_Culture_Sport, Environment
abu_sayyaf	2018-01-02	2021-12-31	Human_Rights, Politics, Religion
action_news_jax	2018-01-01	2021-12-31	Art_Culture_Sport
afghan_refugees	2018-01-01	2021-12-31	Human_Rights
afghanistan_pakistani_militant	2018-01-02	2021-12-31	Human_Rights, Politics, Religion
afghanistan_war	2018-01-02	2022-01-01	Human_Rights, Politics, Religion
afp_paedophile_ring	2018-08-15	2021-11-02	Human_Rights
agent_skripal_spy	2018-03-05	2021-12-29	Politics
aids_hiv	2018-01-02	2021-12-31	Social, Health
al_aqsa_jerusalem_raid	2018-01-15	2021-12-20	Human_Rights, Politics, Religion, Social
alaska_pipeline	2018-01-02	2021-12-31	Economy, Environment
alex_jones	2018-01-02	2021-12-31	Art_Culture_Sport, Politics, Social
alshabab_mogadishu_somalia	2018-01-24	2021-12-31	Human_Rights, Politics, Religion
aluminium_steel_tariffs	2018-01-11	2021-12-31	Economy, Labor, Politics
andhra_pradesh_uttarandhra	2018-02-05	2021-12-03	Economy, Politics, Social
animal_conservation	2018-01-01	2021-12-31	Environment
animal_cruelty	2018-01-02	2021-12-31	Environment, Health
animal_sanctuary_tiverton	2018-03-19	2021-12-18	Environment, Labor
antarctic_ice_melting	2018-01-02	2021-12-31	Environment, Social
antisemitic_jewish_orthodox	2018-02-02	2021-12-26	Human_Rights, Religion, Social
apc_pdp_sheriff	2018-01-03	2021-12-30	Politics
armenia_azerbaijan_border	2018-01-09	2021-12-31	Politics, Social
arvind_kejriwal	2018-01-02	2020-12-31	Human_Rights, Politics, Social
ashland_fundraising	2018-01-03	2021-12-31	Art_Culture_Sport, Economy, Social
asian_hate	2018-01-01	2021-12-31	Human_Rights, Social
aung_san_suu_kyi	2018-01-02	2021-12-31	Human_Rights, Politics, Social
australian_refugees	2018-01-02	2022-01-01	Human_Rights, Labor, Social
baghdad_shiites	2018-01-04	2021-11-29	Religion, Social
ballistic_missile_test	2018-01-01	2021-12-31	Environment, Politics, Social, Tech_Sci
band_debut_album	2018-01-01	2021-12-31	Art_Culture_Sport
benghazi_libya_militias	2018-01-04	2021-12-21	Human_Rights, Politics, Religion
bilateral_cooperation	2018-01-01	2021-12-31	Economy, Politics
birds_invasive_population_species_conservation	2018-01-05	2021-12-31	Environment
black_racism	2018-01-01	2021-12-31	Human_Rights, Social
blacklivesmatter	2019-05-21	2021-12-31	Human_Rights, Social
blue_whale_challenge	2018-01-01	2021-12-31	Social, Health
boat_sinks_die	2018-01-01	2021-12-25	Human_Rights, Social
boeing_737_max_crash	2018-01-01	2021-12-31	Social
boko_haram	2018-01-02	2022-01-01	Human_Rights, Politics, Religion
bollywood_celebrities	2018-01-01	2021-12-31	Art_Culture_Sport
bolsonaro_brazil	2018-01-03	2021-12-31	Human_Rights, Politics, Social
bomber_commits_suicide	2018-01-24	2021-12-14	Social
boris_hunt_tory_debate	2018-01-31	2021-09-04	Politics, Social
boris_johnson	2018-01-01	2021-12-31	Politics
bowe_bergdahl	2018-01-03	2021-12-26	Human_Rights
brain_cells_tumour	2018-01-03	2021-12-31	Social, Tech_Sci, Health
breast_cancer	2018-01-01	2021-12-31	Social, Health
britain_bridge_collapse	2018-01-03	2021-12-23	Art_Culture_Sport, Environment
bsf_jammu_kashmir	2018-01-02	2021-12-31	Politics
buckingham_palace	2018-01-02	2021-12-31	Art_Culture_Sport, Politics, Social
burqa	2018-01-01	2021-12-31	Human_Rights, Religion, Social
bus_accident	2018-01-01	2021-12-31	Labor, Social

california_wildfire	2018-01-01	2021-12-31	Environment
cameron_outcome_referendum	2018-01-02	2021-12-20	Politics, Social
capital_punishment	2018-01-01	2021-12-31	Human_Rights, Social
cathedral_notre_dame	2018-01-02	2021-12-31	Art_Culture_Sport, Environment, Social
charlie_hebdo	2018-01-02	2021-12-31	Art_Culture_Sport, Human_Rights, Politics, Religion, Social
charlottesville_rally_unite	2018-01-02	2021-12-31	Human_Rights, Social
chemtrails	2018-01-01	2021-12-31	Environment, Social, Tech_Sci
climate_warming	2018-01-01	2021-12-31	Environment, Social
co2_emissions	2018-01-01	2021-12-31	Environment, Politics, Tech_Sci
coach_k	2018-01-01	2021-12-31	Art_Culture_Sport
colombia_farc	2018-01-01	2021-12-31	Politics
colorado_shooting	2018-01-02	2021-12-31	Social
confederate_statue_removed	2018-01-04	2021-12-31	Art_Culture_Sport, Human_Rights, Social
contest_nobel_prize_winner	2018-01-17	2021-12-16	Art_Culture_Sport, Tech_Sci
correctional_prisons	2018-01-02	2021-12-31	Human_Rights
crypto_currency_exchange	2018-01-02	2021-12-31	Economy, Labor, Tech_Sci
cuban_embargo	2018-01-02	2021-12-31	Economy, Labor, Politics
cultural_heritage	2018-01-02	2021-12-31	Art_Culture_Sport, Environment, Human_Rights, Religion, Social
cyberbullying	2018-01-01	2021-12-31	Social, Tech_Sci
cybersecurity	2018-01-01	2021-12-31	Politics, Social, Tech_Sci
cybersquatting	2018-01-09	2021-12-23	Economy, Labor, Social, Tech_Sci
dakota_pipeline	2018-01-01	2021-12-31	Economy, Environment
dakota_standing_rock	2018-01-01	2021-12-31	Art_Culture_Sport, Environment, Human_Rights
delhi_pollution	2018-01-01	2021-12-31	Environment, Social, Tech_Sci
democracy_threat	2018-01-01	2021-12-31	Politics, Social
democrat_min_wage	2021-01-02	2021-12-31	Economy, Human_Rights, Labor, Politics
dieselgate	2018-01-01	2021-12-31	Economy, Environment, Labor, Tech_Sci
diplomatic_immunity	2018-01-01	2021-12-31	Politics
divorce_equality	2018-01-01	2021-12-31	Economy, Politics, Social
draft_nfl	2018-01-02	2021-12-31	Art_Culture_Sport
duncan_dallas_ebola	2018-04-12	2021-08-01	Social, Health
duterte_philippines	2018-01-02	2021-01-01	Human_Rights, Politics, Social
e-cigarettes	2018-01-02	2021-12-31	Economy, Environment, Tech_Sci, Health
early_late_voter	2018-01-03	2021-12-31	Politics, Social
earthquake_nepal	2019-01-02	2022-01-01	Environment
efcc_alleged_fraud	2018-01-03	2021-12-30	Economy, Labor
el_chapo_guzman	2018-01-02	2021-12-31	Economy, Social, Health,
elon_musk_tesla	2018-01-02	2021-12-31	Economy, Environment, Labor, Tech_Sci
endangered_species	2018-01-01	2021-12-31	Environment, Tech_Sci, Health
epa_effort	2018-01-03	2021-12-30	Environment, Social, Health
erdogan_coup_d_etat_attempt	2018-01-21	2021-12-06	Politics, Social,
erdogan_turkey	2018-01-01	2021-12-31	Human_Rights, Politics
eruption_volcanic_ash	2018-01-02	2021-12-31	Environment
european_commission	2018-01-01	2021-12-31	Economy, Labor, Politics, Social
fact-checking	2018-01-01	2021-12-31	Social
factory_farming	2018-01-01	2021-12-31	Economy, Environment, Labor
fadnavis_maharashtra	2018-01-02	2021-12-31	Environment, Politics, Social
farmers_irrigation_scheme	2018-01-02	2021-12-31	Economy, Environment, Labor, Tech_Sci
fashion_runway	2018-01-01	2021-12-31	Art_Culture_Sport
fiscal_cuts	2018-01-01	2021-12-31	Economy, Labor, Politics
flat_earth	2018-01-01	2021-12-31	Environment, Social, Tech_Sci
football_galbraith	2019-01-07	2021-12-27	Art_Culture_Sport
ford_kavanaugh	2018-02-13	2021-12-30	Human_Rights, Social
forest_wildfire	2018-01-02	2021-12-31	Environment
garda_dublin	2018-01-03	2021-12-31	Labor, Social
gay_marriages_ban	2018-01-05	2021-12-30	Human_Rights, Politics, Social
gdpr	2018-01-02	2022-01-01	Human_Rights, Politics, Social
geert_wilders_netherlands	2018-01-03	2021-12-05	Human_Rights, Politics, Religion
gender_bathroom	2018-01-01	2021-12-31	Social
gender_gap	2018-01-01	2021-12-31	Economy, Human_Rights, Labor, Politics, Social

gender_identity	2018-01-02	2021-12-31	Human_Rights, Social
george_bush	2018-01-02	2021-12-31	Politics
germany_nazi_merkel	2018-01-02	2021-12-24	Human_Rights, Politics, Religion, Social
grace_mugabe	2018-01-02	2021-12-30	Economy, Environment, Human_Rights, Politics
greek_bailout_tsipras	2018-01-08	2021-07-16	Economy, Labor, Politics, Social
hackers_disinformation	2018-01-03	2021-12-28	Social, Tech_Sci
haftar_lybia	2018-01-02	2021-12-31	Politics
hajj_pilgrimage	2018-01-02	2021-12-31	Religion, Social
halifax_mass_shooting	2018-01-25	2021-12-06	Social
hamas	2018-01-01	2021-12-31	Human_Rights, Politics, Religion
harvey_weinstein_sexual_abuse	2018-01-02	2021-12-31	Art_Culture_Sport, Human_Rights
hate_speech	2018-01-01	2021-12-31	Social
hezbollah_lebanon	2018-01-02	2021-12-31	Human_Rights, Politics, Religion, Social
hijab_ban	2018-01-01	2021-12-31	Human_Rights, Religion, Social
holocaust	2018-01-01	2021-12-31	Human_Rights, Religion, Social
homeless_shelter	2018-01-01	2021-12-31	Human_Rights, Social, Health
hong_kong_protest	2018-01-02	2021-12-31	Human_Rights, Politics, Social
honolulu_civil_beat	2021-11-02	2021-12-31	Art_Culture_Sport, Labor, Politics, Social
houthi_yemen	2018-01-02	2021-12-31	Politics, Religion, Social
humanitarian_aid	2018-01-01	2021-12-31	Human_Rights
hurricane_dorian	2021-01-02	2021-12-31	Environment
hydrogen_vehicles	2018-01-01	2021-12-31	Economy, Environment, Labor, Tech_Sci
illegal_immigration	2018-01-01	2021-12-31	Human_Rights, Politics
imran_khan	2018-01-02	2021-12-31	Art_Culture_Sport, Politics, Social
india_foreign_investment	2018-01-02	2021-12-31	Economy, Labor, Politics
intensive_animal_farming	2018-01-04	2021-12-31	Economy, Environment, Labor
iran_foreign_minister_zarif	2018-01-02	2021-12-13	Politics
iraqi_kurdish_mosul	2018-01-03	2021-12-27	Politics, Religion, Social
ireland_sinn_fein	2018-01-01	2021-12-31	Politics
jakarta_flood	2018-01-03	2021-12-27	Environment
jamal_khashoggi	2018-01-01	2021-12-31	Art_Culture_Sport, Human_Rights, Politics
jeffrey_epstein	2018-01-01	2021-12-31	Human_Rights
jeremy_corbyn_labour	2018-01-02	2021-12-31	Labor, Politics, Social
john_mccain	2018-01-01	2021-12-31	Politics
joyce_marcel	2018-01-04	2021-12-31	Art_Culture_Sport
julian_assange_wikileaks	2018-01-02	2021-12-31	Art_Culture_Sport, Economy, Politics, Social
kabila_congo	2018-01-01	2021-12-31	Politics
karnataka_assembly_poll	2018-01-01	2021-12-30	Politics
kayapo	2018-01-03	2021-12-31	Environment, Human_Rights
kenney_elected_mayor_philadelphia	2018-01-20	2021-12-31	Politics, Social
kiev_donetsk_separatists	2018-01-13	2021-12-31	Politics, Social
kiir_machar_south_sudan	2018-01-03	2021-12-31	Politics
kilauea_eruption	2018-01-01	2021-12-31	Environment, Tech_Sci
kim_jong_un	2018-01-01	2021-12-31	Human_Rights, Politics
klopp_liverpool	2018-01-02	2021-12-31	Art_Culture_Sport
labor_movement	2018-01-02	2021-12-31	Economy, Labor, Politics
lahore_rape	2018-01-01	2021-12-26	Human_Rights
lee_kuala_lumpur	2018-01-02	2021-12-31	Social
legalize_prostitution	2018-01-05	2021-12-30	Social
leo_varadkar_taoiseach	2018-01-02	2021-12-31	Human_Rights, Politics, Social
lgbt_discrimination	2018-01-02	2021-12-31	Human_Rights, Social
london_mayor_re-election_bid	2018-02-01	2021-11-15	Politics, Social
louisiana_parish_arrested	2018-01-02	2021-12-31	Social
lung_cancer	2018-01-01	2021-12-31	Social, Tech_Sci, Health
macron_france	2018-01-01	2021-12-31	Politics
maduro_venezuela	2018-01-02	2021-12-29	Politics
marco_rubio_debate	2021-01-03	2021-12-25	Politics
marijuana_legalization	2018-01-02	2022-01-01	Economy, Politics, Social, Health
marine_corps	2018-01-01	2021-12-31	Labor, Social

marine_le_pen	2018-01-02	2021-12-31	Politics
mars_mission	2018-01-01	2021-12-31	Environment, Tech_Sci
mars_spacecraft_mission	2018-01-02	2021-12-31	Environment, Tech_Sci
martin_luther_king	2018-01-02	2022-01-01	Human_Rights, Politics, Social
maryam_nawaz	2018-01-01	2021-12-31	Politics
mass_shootings	2018-01-02	2022-01-01	Social
measles	2018-01-01	2021-12-31	Social, Tech_Sci, Health
meghan_harry	2018-01-02	2022-01-01	Politics, Social
merkel_germany	2018-01-01	2021-12-31	Politics
metoo	2018-01-01	2021-12-31	Human_Rights, Labor, Social
metric_tonnes_waste	2018-01-02	2021-12-30	Environment, Tech_Sci
mexican_migrants	2018-01-01	2021-12-31	Human_Rights, Politics, Social
mexico_wall	2018-01-01	2021-12-31	Human_Rights, Politics, Social
mh370	2019-01-02	2021-12-29	Social
michael_brown_shooting	2018-01-03	2021-12-31	Human_Rights, Social
michael_cohen_lawyer	2018-01-05	2021-12-31	Politics
migration_pact	2018-01-05	2021-12-31	Human_Rights, Politics, Social
mike_pence_indiana	2018-01-02	2021-12-20	Politics
mindanao_martial_law	2018-01-01	2021-12-31	Human_Rights, Politics, Social, Health
minimum_wage	2018-01-02	2021-12-31	Economy, Human_Rights, Labor, Politics, Social
mission_moon	2018-01-01	2021-12-31	Environment, Tech_Sci
modis_narendra	2018-01-05	2021-12-29	Politics
morsi_sisi_egypt	2018-01-09	2021-12-30	Politics, Religion
mueller_probe	2018-01-02	2021-12-30	Politics
muslim_brotherhood	2018-01-01	2021-12-31	Religion
nafta_trade	2018-01-01	2021-12-31	Economy, Labor, Politics
native_american_indigenous	2018-01-01	2021-12-31	Human_Rights
natural_gas_prices	2018-01-01	2021-12-31	Economy, Environment, Labor, Politics
nauru_refugees	2018-01-02	2021-12-31	Human_Rights
nelson_bay_cup	2018-01-13	2021-12-31	Art_Culture_Sport
netanyahu	2018-01-01	2021-12-31	Politics
nicola_sturgeon_scotland_independence	2018-01-02	2021-12-31	Politics, Social
nigel_farage_ukip	2018-01-02	2021-12-28	Politics, Social
nikolas_cruz	2018-01-09	2021-12-31	Social
nitish_kumar_bihar	2018-01-02	2021-12-31	Politics
npa_rebels	2018-01-01	2021-12-31	Politics
nuclear_war	2018-01-01	2021-12-31	Environment, Politics, Social, Tech_Sci
obrador_mexico	2018-01-01	2021-12-31	Politics
ocean_fishing	2018-01-02	2021-12-31	Economy, Environment, Labor
off_peak_season_travel	2018-01-01	2021-12-31	Art_Culture_Sport, Economy, Environment
offshore_wind	2018-01-01	2021-12-31	Economy, Environment, Tech_Sci
operation_varsity_blues	2018-06-16	2021-12-31	Art_Culture_Sport, Labor, Social
opioid_drug_crisis	2018-01-01	2021-12-31	Social, Health
organ_trade	2018-01-01	2021-12-31	Economy, Human_Rights, Health
pacific_solution	2018-01-01	2021-12-31	Human_Rights, Politics
panama_papers	2018-01-02	2021-12-31	Economy
paul_manafort	2018-08-02	2021-12-30	Politics
pension_retirement_age	2018-01-01	2021-12-31	Economy, Labor, Politics, Social, Health
pkk	2018-01-02	2021-12-31	Human_Rights, Politics, Social
planned_pregnancy	2018-01-02	2022-01-01	Human_Rights, Social, Tech_Sci, Health
plastic_surgery	2018-01-02	2021-12-31	Social, Tech_Sci, Health
ponte_morandi	2018-01-03	2021-12-31	Social
portland_standoff	2018-01-03	2021-12-12	Human_Rights, Social
protest_sign_mou	2018-01-09	2021-12-22	Social
rajapaksa_sri_lanka	2018-01-02	2021-12-31	Politics
rajasthan_mps	2018-01-07	2021-12-31	Politics
rajya_sabha_elections_bjp	2018-01-02	2021-12-30	Politics, Social
rakhine_rohingya_myanmar	2018-01-01	2021-12-31	Human_Rights, Religion, Social
ramaphosa_south_africa	2018-01-01	2021-12-31	Human_Rights, Labor, Politics, Social
randolph_holhut	2018-02-21	2021-09-30	Art_Culture_Sport

ransomware	2018-01-01	2021-12-31	Economy, Tech_Sci
rauner_illinois	2018-01-01	2021-12-22	Politics
recreational_cannabis	2018-01-01	2021-12-31	Politics, Social, Health
religion_freedom	2018-01-01	2021-12-31	Human_Rights, Religion
reynolds_mourned	2018-07-04	2021-12-31	Labor, Social
rock_n_roll_savannah_marathon	2018-01-04	2021-12-14	Art_Culture_Sport, Social
roe_v._wade_case	2018-01-02	2021-12-31	Human_Rights, Social
ryanair_pilot_strike	2018-01-04	2019-10-03	Labor
salman_saudi_arabia	2018-01-01	2021-12-31	Human_Rights, Politics, Religion,
santos_colombia	2018-01-02	2021-12-31	Politics
sargsyan_armenia	2018-01-02	2021-12-31	Politics
scientist_hansen_nasa	2018-01-06	2021-12-29	Art_Culture_Sport, Environment, Labor, Tech_Sci
scott_morrison_kabul	2018-02-18	2021-12-30	Human_Rights, Politics
scott_walker_wisconsin	2018-01-02	2021-12-31	Politics
self-driving_car	2018-01-01	2021-12-31	Economy, Labor, Social, Tech_Sci
shiv_sena_maharashtra	2018-01-02	2021-12-31	Human_Rights, Politics
sirisena_sri_lanka	2018-01-01	2021-12-30	Politics
smart_cities	2018-01-01	2021-12-31	Economy, Environment, Social, Tech_Sci
smith_scandal_resigns	2018-03-25	2021-11-26	Art_Culture_Sport
snowden	2018-01-02	2021-12-31	Human_Rights, Politics, Social, Tech_Sci
society_civil_servants	2018-01-01	2021-12-31	Labor, Politics
solar_panels	2018-01-01	2021-12-31	Economy, Environment, Tech_Sci
sonia_rahul_gandhi	2018-01-02	2021-12-31	Politics
spacex_moon_mission	2018-01-01	2021-12-30	Environment, Labor, Tech_Sci
spending_cuts	2018-01-01	2021-12-31	Economy, Labor, Politics, Social
stolen_identity	2018-01-01	2021-12-31	Social
syria_kurdish_war	2018-01-02	2021-12-30	Human_Rights, Politics, Social
syrian_refugees	2018-01-01	2021-12-31	Human_Rights
tamil_ltte	2018-01-04	2021-12-31	Human_Rights, Politics, Social
telangana_rao_trs	2018-01-02	2021-12-31	Politics
theresa_may_brexit	2018-01-02	2021-12-30	Politics, Social
tiger_woods_win	2018-01-02	2021-12-31	Art_Culture_Sport
tony_abbott_malcolm_turnbull	2018-01-02	2022-01-01	Politics
tourism_boost	2018-01-01	2021-12-31	Economy, Environment, Labor
tourists_overrun	2018-01-03	2021-12-31	Environment, Social
tpp	2018-01-02	2021-12-31	Economy, Politics
truck_highway_crashes	2018-01-02	2021-12-31	Labor
trudeau_canada	2018-01-02	2021-12-31	Politics
trump_impeachment	2018-01-01	2021-12-31	Politics
tsai_ing-wen_taiwan	2018-01-01	2021-12-31	Human_Rights, Politics, Social
tshisekedi_congo	2018-01-01	2021-12-31	Politics
tupac	2018-01-26	2021-12-09	Art_Culture_Sport
uber_ride_sharing	2018-01-02	2021-12-31	Economy, Labor
uhuru_kenyatta	2018-01-01	2021-12-31	Politics
undercover_comey_fbi	2018-02-07	2021-11-08	Politics
undp_procurement	2018-01-04	2021-12-31	Economy, Human_Rights, Labor, Politics, Social
unhcr_refugees	2018-01-01	2021-12-31	Human_Rights
vatican_abuse	2018-01-03	2022-01-01	Human_Rights, Religion
white_supremacist	2018-01-01	2021-12-31	Human_Rights, Social
william_barr_attorney_general	2018-04-30	2021-12-31	Politics
william_kate_middleton	2018-01-02	2021-12-31	Politics, Social
williams_plead_guilty	2018-01-03	2021-12-25	Social
wirecard_scandal	2019-01-02	2022-05-05	Economy, Labor
women_abortion	2018-01-02	2022-01-01	Human_Rights, Social, Health
xi_jinping	2018-01-01	2021-12-31	Politics
xinhua_silk_road	2018-01-19	2021-12-30	Economy, Labor
yakubu_dogara_sacked	2018-01-15	2021-12-05	Politics
yanukovych_crimea	2018-01-03	2021-12-30	Politics
ywca	2018-01-02	2021-12-31	Human_Rights, Religion, Social
zayed_uae	2018-01-01	2021-12-31	Economy, Politics, Social
zika_virus	2018-01-02	2021-12-31	Social, Health
zuma_south_africa	2018-01-01	2021-12-31	Human_Rights, Politics, Social

Table 1. List of terms employed to perform the research for each topic together with the first and last date when a post related to each topic was found.

Data breakdown at topic level

Evaluating the relationship between topic engagement evolution and controversy

Fig S1. Correlation between α and LH score for each identified topic.

Goodness of the fitting procedure

We fit the cumulative evolution of engagement for the topics in the List of topics employed Section with the function $f_{\alpha,\beta}$. The fitting procedure produces, for each topic i, the tuples $\left(\hat{\alpha_i}, \hat{\beta_i}\right)$ and $\left(SE(\hat{\alpha_i}), SE(\hat{\beta_i})\right)$ containing the estimated parameters with their standard errors, respectively. Fig. S2 provides a joint distribution of the errors $\left(SE(\hat{\alpha_i}), SE(\hat{\beta_i})\right)$ for each topic in relationship with the number of posts they produced. We observe how $SE(\hat{\alpha_i})$ errors follow a log-normal distribution, while $SE(\hat{\beta_i})$ errors have a normal one. We can observe a reduction in the errors for both parameters as the number of posts per topic increases. We formerly assess such relationship by computing a Spearman correlation coefficient between each standard error and the number of posts per topic, obtaining a value of $\rho(SE(\hat{\alpha_i}), posts_i) = -0.44$ and $\rho(SE(\hat{\beta_i}), posts_i) = -0.25$. We can therefore conclude that our fitting procedure provides results with a reducing error as the number of observations increases.

Fig S2. Joint distribution of the errors $SE(\hat{\alpha_i})$ and $SE(\hat{\beta_i})$ for each topic i, whose cumulative curve was estimated by means of $f_{\alpha,\beta}$. The colour of each point represent the number of posts produced by topic i.

Assessing the differences of engagement behaviors across topic categories

**Category**	α	β	**SI**
Art_Culture_Sport	0.043 (0.1647)	693.8 (236)	0.49 (0.13)
Economy	0.0045 (0.0022)	752.36 (241.45)	0.48 (0.14)
Environment	0.0215 (0.1214)	761.41 (207.81)	0.47 (0.11)
Human_Rights	0.0244 (0.113)	765.48 (223.24)	0.47 (0.14)
Labor	0.0137 (0.0618)	715.41 (286.41)	0.49 (0.15)
Politics	0.0192 (0.0953)	711.78 (243.12)	0.5 (0.15)
Religion	0.0405 (0.1906)	786.5 (184.07)	0.46 (0.12)
Social	0.024 (0.1182)	728.58 (204.3)	0.49 (0.12)
Tech_Sci	0.004 (0.0013)	801.76 (187.67)	0.46 (0.11)
Health	0.0045 (0.0016)	692.03 (128.7)	0.52 (0.08)

Table 2. Summary of α, β and Speed Index mean values (and SD) per topic category.

Category	Rho	p.value
All	-0.26	0
Art_Culture_Sport	-0.2	0.2631
Economy	-0.2	0.1665
Environment	-0.21	0.1379
Human_Rights	-0.29	0.0067
Labor	-0.35	0.0162
Politics	-0.36	0
Religion	-0.12	0.5308
Social	-0.23	0.0057
Tech_Sci	-0.21	0.2256
Health	-0.45	0.0267

Table 3. Spearman's Rho between Speed and Love-Hate Score per category (CI = 0.95). For readability, 0 represents values lower than 0.0001.

	A_C_S	Econ	Env	H_R	Labor	Politics	Religion	Social	Tech	Health
**A_C_S**		0.3621	0.9226	0.3671	0.7594	0.5024	0.8931	0.8646	0.1195	0.9688
**Econ**	0.3621		0.2994	0.0104	0.4292	0.0146	0.3788	0.0793	0.3247	0.3496
**Env**	0.9226	0.2994		0.1287	0.9135	0.2025	0.992	0.5873	0.0546	0.9688
**H_R**	0.3671	0.0104	0.1287		0.134	0.6761	0.2437	0.2236	**0.0009**	0.2014
**Labor**	0.7594	0.4292	0.9135	0.134		0.1818	0.8575	0.4937	0.0874	0.9322
**Politics**	0.5024	0.0146	0.2025	0.6761	0.1818		0.335	0.3465	0.0018	0.2755
**Religion**	0.8931	0.3788	0.992	0.2437	0.8575	0.335		0.6836	0.0976	0.9347
**Social**	0.8646	0.0793	0.5873	0.2236	0.4937	0.3465	0.6836		0.011	0.6151
**Tech**	0.1195	0.3247	0.0546	**0.0009**	0.0874	0.0018	0.0976	0.011		0.08
**Health**	0.9688	0.3496	0.9688	0.2014	0.9322	0.2755	0.9347	0.6151	0.08	

Table 4. p-values of the two-tailed Mann–Whitney U tests performed on the average α parameter value between categories (CI = 0.95). Bold values represent the value for which the null hypothesis was rejected.

	A_C_S	Econ	Env	H_R	Labor	Politics	Religion	Social	Tech	Health
**A_C_S**		0.1688	0.171	0.2124	0.5789	0.7218	0.2055	0.5985	0.0532	0.8082
**Econ**	0.1688		0.952	0.7537	0.6159	0.1358	0.9875	0.181	0.5199	0.0567
**Env**	0.171	0.952		0.718	0.6412	0.0932	0.9759	0.1386	0.4838	0.0315
**H_R**	0.2124	0.7537	0.718		0.6522	0.1274	0.7229	0.1808	0.2939	0.0599
**Labor**	0.5789	0.6159	0.6412	0.6522		0.5506	0.5529	0.7086	0.2948	0.2793
**Politics**	0.7218	0.1358	0.0932	0.1274	0.5506		0.1676	0.7814	0.0274	0.3969
**Religion**	0.2055	0.9875	0.9759	0.7229	0.5529	0.1676		0.1816	0.5318	0.0418
**Social**	0.5985	0.181	0.1386	0.1808	0.7086	0.7814	0.1816		0.0338	0.2407
**Tech**	0.0532	0.5199	0.4838	0.2939	0.2948	0.0274	0.5318	0.0338		0.0076
**Health**	0.8082	0.0567	0.0315	0.0599	0.2793	0.3969	0.0418	0.2407	0.0076	

Table 5. p-values of the two-tailed Mann–Whitney U tests performed on the average β parameter value between categories (CI = 0.95). Bold values represent the value for which the null hypothesis was rejected.

	A_C_S	Econ	Env	H_R	Labor	Politics	Religion	Social	Tech	Health
**A_C_S**		0.4121	0.3312	0.3731	0.5789	0.869	0.4838	0.8942	0.1862	0.4756
**Econ**	0.4121		0.9253	0.9877	0.9856	0.2284	0.9212	0.3115	0.5664	0.0496
**Env**	0.3312	0.9253		0.8325	0.908	0.1166	0.9037	0.1748	0.6437	0.0223
**H_R**	0.3731	0.9877	0.8325		0.9216	0.1215	0.9618	0.2021	0.5007	0.0447
**Labor**	0.5789	0.9856	0.908	0.9216		0.3042	0.8918	0.4106	0.5814	0.0888
**Politics**	0.869	0.2284	0.1166	0.1215	0.3042		0.2611	0.7012	0.0617	0.3407
**Religion**	0.4838	0.9212	0.9037	0.9618	0.8918	0.2611		0.3424	0.6332	0.0733
**Social**	0.8942	0.3115	0.1748	0.2021	0.4106	0.7012	0.3424		0.0784	0.1822
**Tech**	0.1862	0.5664	0.6437	0.5007	0.5814	0.0617	0.6332	0.0784		0.0103
**Health**	0.4756	0.0496	0.0223	0.0447	0.0888	0.3407	0.0733	0.1822	0.0103	

Table 6. p-values of the two-tailed Mann–Whitney U tests performed on the average Speed Index between categories (CI = 0.95). Bold values represent the value for which the null hypothesis was rejected.

	A_C_S	Econ	Env	H_R	Labor	Politics	Religion	Social	Tech	Health
**A_C_S**		0.0369	0.2472	**0.0001**	0.009	**0.0001**	0.0048	**0.0005**	0.3624	0.0656
**Econ**	0.0369		0.1372	0.0023	0.4167	0.0089	0.0792	0.0501	0.0777	0.9081
**Env**	0.2472	0.1372		**0**	0.0238	**0**	0.004	**0.0003**	0.7374	0.1817
**H_R**	**0.0001**	0.0023	**0**		0.0401	0.4758	0.5167	0.1535	**0**	0.0275
**Labor**	0.009	0.4167	0.0238	0.0401		0.107	0.3184	0.3601	0.0121	0.5761
**Politics**	**0.0001**	0.0089	**0**	0.4758	0.107		0.9014	0.4024	**0**	0.0708
**Religion**	0.0048	0.0792	0.004	0.5167	0.3184	0.9014		0.7623	0.0013	0.1672
**Social**	**0.0005**	0.0501	**0.0003**	0.1535	0.3601	0.4024	0.7623		**0.0002**	0.1763
**Tech**	0.3624	0.0777	0.7374	**0**	0.0121	**0**	0.0013	**0.0002**		0.1064
**Health**	0.0656	0.9081	0.1817	0.0275	0.5761	0.0708	0.1672	0.1763	0.1064	

Table 7. p-values of the two-tailed Mann–Whitney U tests performed on the average Love-Hate Score between categories (CI = 0.95). Bold values represent the value for which the null hypothesis was rejected.

	A_C_S	Env	Tech	Politics	Social	H_R
**A_C_S**				**0.0001**	**0.0003**	**0**
**Env**				**0**	**0.0002**	**0**
**Tech**				**0**	**0.0001**	**0**
**Politics**	0.9999	1	1			
**Social**	0.9998	0.9998	0.9999			
**H_R**	1	1	1			

Table 8. p-values of Mann–Whitney U test on LH mean value between categories for which the null hypotesis was rejected in Table 7 (H1: $\mu_r > \mu_c$, where r and c represent row and column category; Conf. Level = 0.95). Bold values represent the value for which the null hypothesis was rejected.
