# Supplementary figures and images for: Characterizing engagement dynamics across topics on Facebook
Source: PLoS One. 2023 Jun 28;18(6):e0286150. doi: 10.1371/journal.pone.0286150 (PMC10306180; doi:10.1371/journal.pone.0286150)

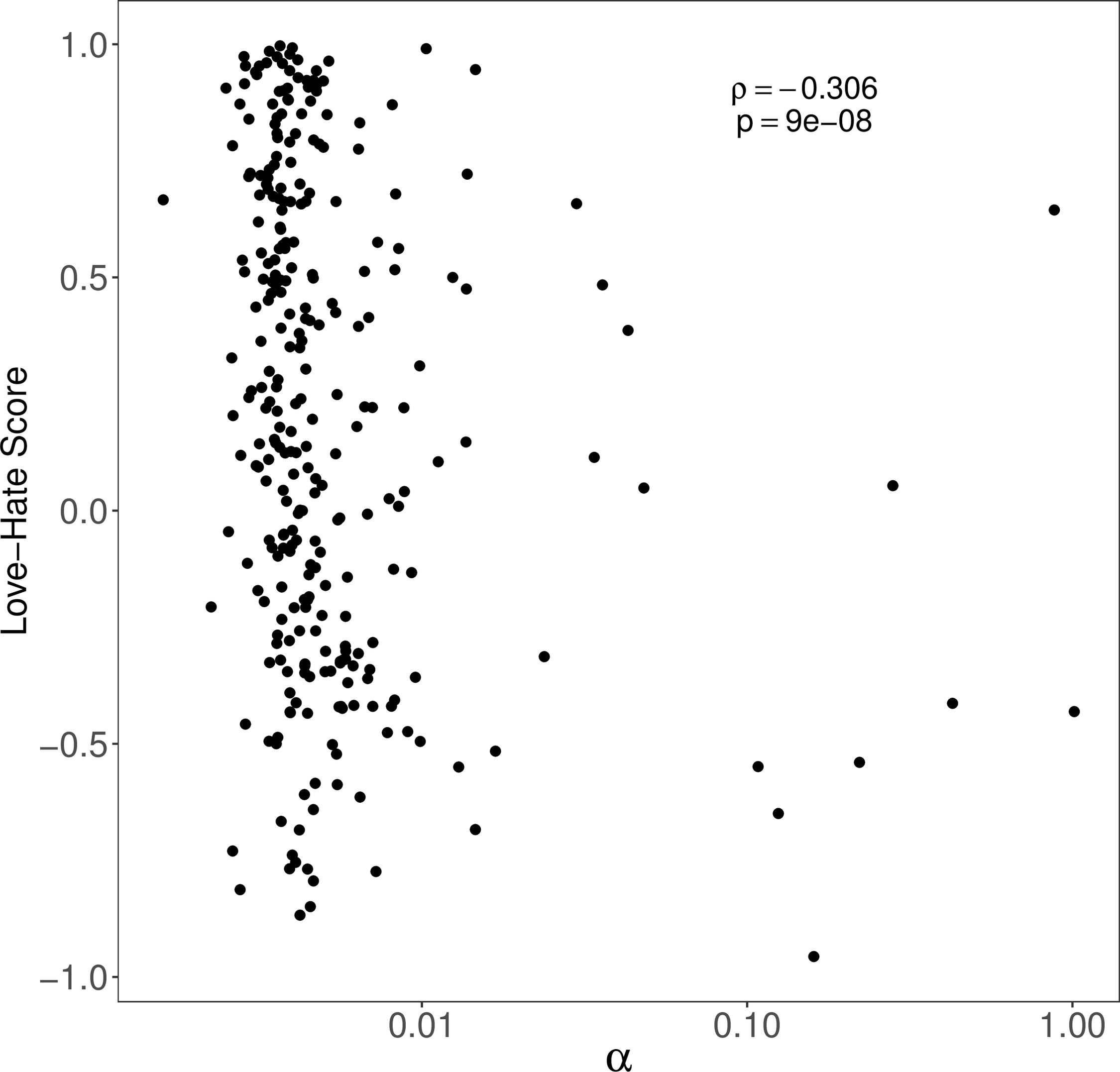

Supplement: S1 Fig — (TIF) [file pone.0286150.s003.tif]

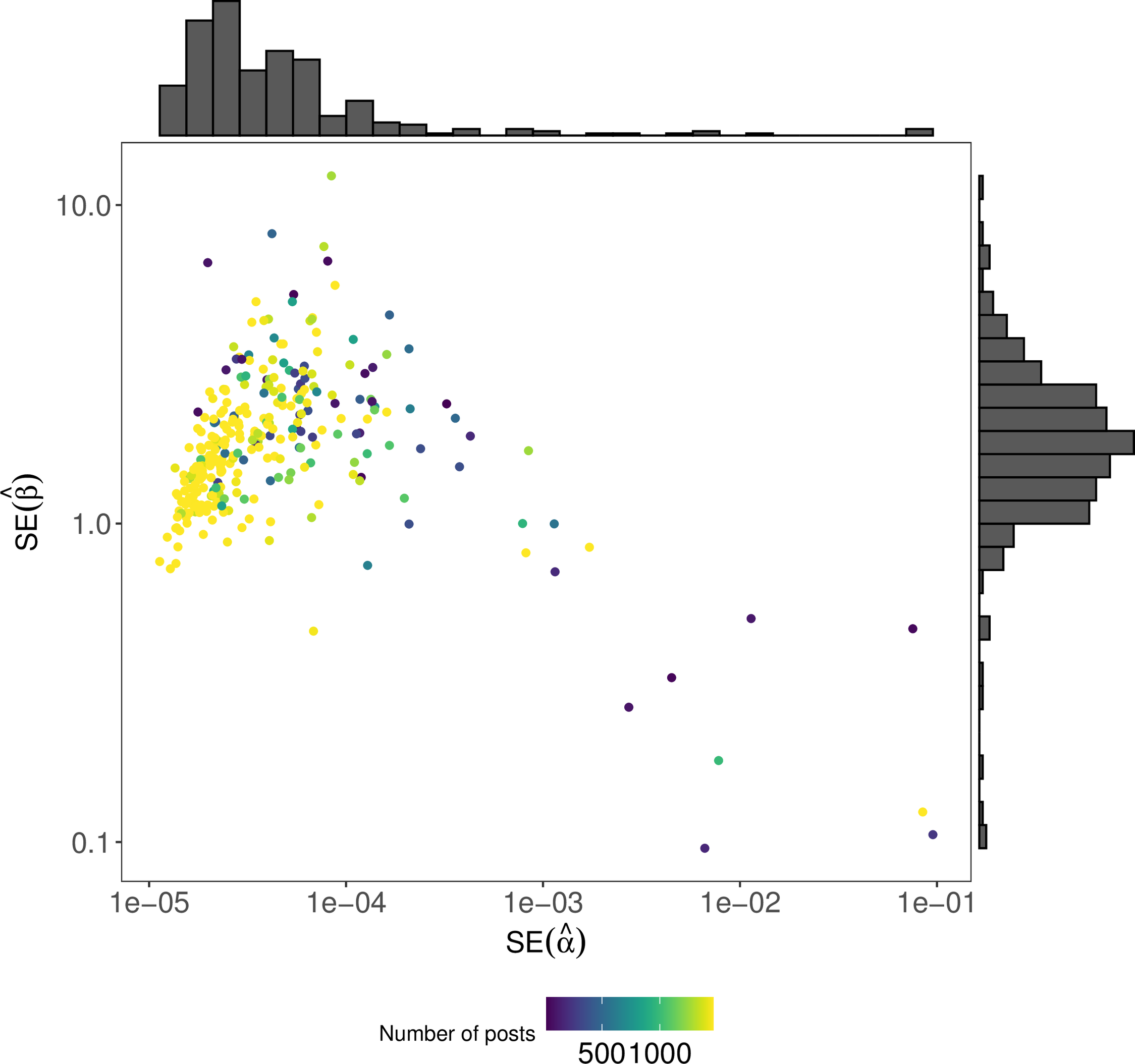

Supplement: S2 Fig — The colour of each point represents the number of posts produced by topic i. (TIF) [file pone.0286150.s004.tif]
